# Electrochemical Characterization of Riboflavin-Enhanced Reduction of Trinitrotoluene

**DOI:** 10.3390/s111110840

**Published:** 2011-11-18

**Authors:** James J. Sumner, Kevin Chu

**Affiliations:** 1 United States Army Research Laboratory, RDRL-SEE-O, 2800 Powder Mill Road, Adelphi, MD 20873, USA; E-Mail: kevin.chu.ctr@mail.mil; 2 Department of Biomedical Engineering, Washington University, Saint Louis, MO 63130, USA

**Keywords:** trinitrotoluene, dinitrotoluene, reduction, riboflavin, bioremediation, AC voltammetry

## Abstract

There is great interest in understanding trinitrotoluene (TNT) and dinitrotoluene (DNT) contamination, detection and remediation in the environment due to TNT’s negative health effects and security implications. Numerous publications have focused on detecting TNT in groundwater using multiple techniques, including electrochemistry. The main degradation pathway of nitrotoluenes in the environment is reduction, frequently with biological and/or photolytic assistance. Riboflavin has also been noted to aid in TNT remediation in soils and groundwater when exposed to light. This report indicates that adding riboflavin to a TNT or DNT solution enhances redox currents in electrochemical experiments. Here AC voltammetry was performed and peak currents compared with and without riboflavin present. Results indicated that TNT, DNT and riboflavin could be detected using AC voltammetry on modified gold electrodes and the addition of riboflavin affected redox peaks of TNT and DNT. Poised potential experiments indicated that it is possible to enhance reduction of TNT in the presence of riboflavin and light. These results were dramatic enough to explain long term enhancement of bioremediation in environments containing high levels of riboflavin and enhance the limit of detection in electrochemically-based nitrotoluene sensing.

## 1. Introduction

There is great interest in understanding trinitrotoluene (TNT) and dinitrotoluene (DNT) contamination, detection and remediation in the environment due to TNT’s negative health effects as well as security implications [1,2]. Current protocols used for field analysis include colorimetric and immunoassay techniques that require skilled technicians collecting samples and processing them in a controlled environment for the best results [3,4]. The protocols are not particularly sensitive as they are only designed to detect the drinking water health advisory level of 2,000 ppm however, these procedures are costly to implement due to the fact they take significant amounts of time as samples must be collected in the field and transported to a laboratory for analysis. Electrochemistry offers and interesting alternative to other methods as instruments are traditionally low-cost, rugged, and easily engineered to be portable. Modern blood glucose monitors are an excellent example of these characteristics. Nitroaromatics have been studied using electrochemistry extensively over the past few years with multiple published techniques to analyze DNT and TNT with limits of detection near 10 ppb [5–10]. While this is of interest, to compete with high performance liquid chromatographic techniques for analysis of environmental samples of nitroaromatic compounds the limit of detection needs to be decreased by another one to four orders of magnitude [11–13].

Remediation is another large area of interest when studying nitroaromatics in the environment. Techniques studied have included bioremediation, photocatalysis and electrochemistry [14–19]. Riboflavin, or vitamin B2, has been reported to aid in TNT reduction in remediation experiments set to simulate soils and groundwater [20–22]. These experiments employed riboflavin spiked into reaction vessels exposed to sunlight or broadband UV/visible light sources. This exposure to light as well as riboflavin was required to have a significant enhancement of TNT reduction. While the electrochemical characterization of riboflavin has been examined to a lesser extent, recent publications validate the electrochemistry studied here [23,24]. The enhancement of the electrochemical signal of riboflavin could also be useful in studying this coenzyme composition for its effects on metabolism, nutrition and as a photosensitive agent [24,25].

It was postulated that TNT redox activity should be able to be enhanced by adding riboflavin and exposing it to a broad band light source. This would be useful to understand mechanisms in the simulated environmental samples reported in the remediation literature [20–22] as well as provide a means to increase the sensitivity of electrochemically-based sensors for nitroaromatics.

Experiments were performed using AC voltammetry and comparing peak currents with and without riboflavin as well as exposure to broadband light. Results indicated that DNT, TNT and riboflavin could be detected independently using AC voltammetry on self-assembled monolayer (SAM), modified gold electrodes. The exposure of nitroaromatic analytes to riboflavin and light affected redox peaks of TNT and DNT. Poised potential experiments were also performed in the presence of riboflavin and light to demonstrate that it is possible to enhance reduction of TNT over time. These results were dramatic enough to help explain long term enhancement of remediation of TNT in environments containing high levels of riboflavin as well as a way to enhance the limit of detection of electrochemically-based TNT sensors.

## 2. Experimental Section

### 2.1. Materials and Instrumentation

The electrolyte was sodium perchlorate, obtained from Aldrich, prepared as a 1 M aqueous solution. Stocks of the analytes 2,4,6-trinitrotoluene (Chem Service, West Chester, PA, USA) and 2,4-dinitrotoluene (Sigma-Aldrich, St. Louis, MO, USA) were prepared at a concentration of 10,000 ppm in actetonitrile. Those stocks and riboflavin (Sigma-Aldrich, St. Louis, MO, USA) were diluted in the electrolyte in concentrations from 10 to 1,000 ppb for analysis. Thiol-terminated alcohols (mercaptobenzoic acid, cystamine, mercaptoethanol, mercaptopropanol and mecaptohexanol, all from Aldrich) for self-assembled monolayer (SAM) formation on the gold electrodes were prepared as 1 mM ethanolic solutions. Electrodes were voltammetrically stripped prior to SAM formation using 0.5 M sulfuric acid diluted from concentrated stock obtained from Fisher and then they were polished sequentially with 1.0, 0.3 and 0.05 micron alumina, sonicating after each polishing step. All water was purified using a Barnstead EasyPure RF to 18 MΩ cm.

The electrochemical cell was a three electrode configuration using a 2 mm diameter SAM-modified gold disk electrode as the working electrode, Ag/AgCl reference electrode, and platinum wire counter electrode all obtained from CH Instruments (Austin, TX, USA). All electrochemical experiments were carried out using a CH Instruments model 660a electrochemical workstation. Broadband light was provided by a 75 W 120 V halogen bulb placed 30 cm from the electrochemical cell.

### 2.2. Methods

SAM-modified electrodes were prepared by voltammetric stripping and polishing of the electrode followed by soaking in a 1 mM ethanolic solution of the respective mercapto-alcohol overnight. Most procedures used AC voltammetry at 10 Hz with a potential sweep between 0.3 V and −0.8 V. The sweep was performed as 4 mV steps with a measurement time of 2 s per step. A background scan of each electrode was performed in order to verify SAM formation before the addition of TNT or riboflavin. Either TNT or riboflavin was titrated into the electrochemical cell at concentrations from 10 to 1,000 ppb and ac voltammetric sweeps were performed at least three times to insure a stable response. The final experiments on each electrode involved introducing 10–1,000 ppb of the analyte to the solution not previously present (either the TNT or riboflavin) to evaluate any enhancement. In experiments with light exposure, the electrochemical cell was illuminated only during the voltammetric sweep.

Removing the polymer generated by the reduction of nitroaromatics from the modified working electrode was much easier than bare electrodes however it was still a multistep process. It required electrochemical stripping in 0.5 M H_2_SO_4_ sweeping from 0 V to 1.0 V using cyclic voltammetry. After electrochemically stripping the electrode, it was polished sequentially using 1 μm, 0.3 μm, and 0.05 μm alumina sonicating between each step and prior to placing in the ethanolic mercaptohexanol solution for preparation of a fresh SAM.

## 3. Results and Discussion

### 3.1. Electrochemistry of TNT, DNT and Riboflavin

**Scheme 1.**
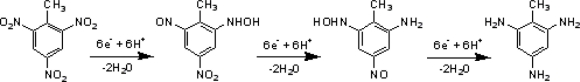
Schematic of the reduction of TNT as a three step process explaining the three peaks generated in voltammograms.

The reduction mechanism of the nitroaromatics, TNT and DNT has been described in previous work [5,7,19]. Both species tend to use the electron deficient nitrogen groups as electron acceptors and upon applied potential will be reduced from nitro, to hydroxylamine and subsequently to amine functionalities. The reaction sequence in Scheme 1 is based on the work of Grigoriants *et al*. described in [7] and agrees with the recent literature [5,19]. This figure shows this reduction of TNT as a three step process to describe the three redox peaks generated in the voltammetry.

When TNT or DNT are reduced and aromatic amines are formed it is possible to further reduce these species to azides. These aromatic azides can then polymerize onto the electrode surface [26–28]. This polymerization blocks the electrode surface and makes obtaining reproducible results difficult as well as makes the surface very difficult to clean. To prevent this issue the working electrode was modified with a self-assembled monolayer. The intent of this monolayer was not only to make the surface easier to clean after nitroaromatic analysis but also to provide a broader potential window in which to scan the gold working electrode. Several monolayers were examined including mercaptobenzoic acid, cystamine, mercaptoethanol, mercaptopropanol and mercaptohexanol. Mercaptohexanol was the only monolayer which packed well enough to protect the electrode surface, however, due to the length it was necessary to run ac voltammetry at very low frequencies (1 to 10 Hz) in order to not outpace the electron transfer kinetics.

**Figure 1.**
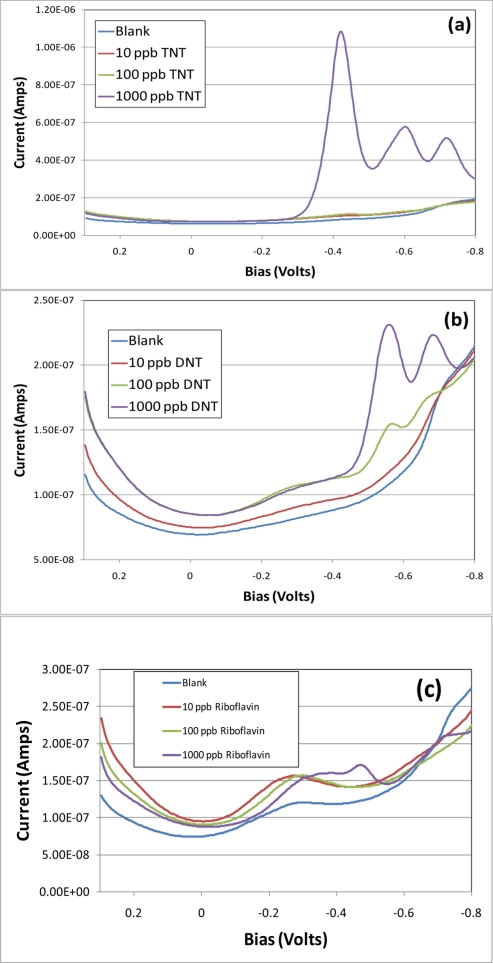
AC voltammograms of the electrode response to titrations of (**a**) TNT, (**b**) DNT and (**c**) riboflavin. The plots show a blank, 10 ppb, 100 ppb and 1,000 ppb of each analyte in 1 M sodium perchlorate electrolyte with a mercaptohexanol SAM-modified working electrode.

All subsequent data shown was collected using a mercaptohexanol modified gold working electrode. Figure 1 shows the ac voltammetry of neat aqueous preparations of (a) TNT, (b) DNT and (c) riboflavin. The frequency was 10 Hz, amplitude 25 mV and the sweep was performed in 4 mV steps. Each analyte was examined independently by first interrogating with a blank followed by titrations to yield total concentrations of 10, 100, and 1,000 ppb in aqueous 1 M sodium perchlorate. Each scan plotted represents an average of three voltammetric sweeps. In this study, when there was no exposure to riboflavin or light, TNT showed three significant redox peaks at 1,000 ppb without background subtraction. DNT was more sensitive showing two distinct redox peaks at 100 ppb. Riboflavin did not exhibit redox activity until concentrations reached 1,000 ppb with a sharp peak at ∼−0.45 V, the dominate structure in these scans of riboflavin is a background peak due to the SAM-modified electrode.

### 3.2. The Effects of Riboflavin on the Electrochemistry of TNT and DNT

**Figure 2.**
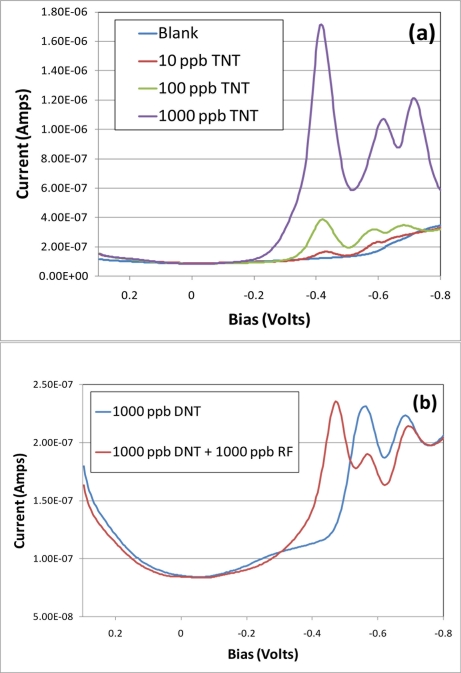
AC voltammograms of the electrode response to the analytes **(a)** TNT (0–1,000 ppb) exposed to 1,000 ppb riboflavin and **(b)** DNT (1,000 ppb) with and without exposure to 1,000 ppb riboflavin. Note that the DNT only produced 2 redox peaks while the 1,000 ppb riboflavin exposed DNT produces a significantly larger peak at ∼0.45 V than that of TNT or riboflavin alone.

With reports in the literature describing riboflavin-enhanced reduction of TNT in simulated ground water and soils [20–22] it was postulated that the effect on electrochemistry should be evaluated. Reports also indicated that the reduction of TNT was further enhanced by exposure to light. A complicating factor to the analysis of enhanced reduction of TNT in this system is that first redox peak for TNT and the redox peak for riboflavin appear at a very similar bias *versus* Ag/AgCl however, Figure 2 demonstrates this enhancement for both (a) TNT and (b) DNT with riboflavin present. These example scans show a titration of TNT or DNT from 0 to 1,000 ppb with the electrochemical cell exposed to light as well as having the working electrode pretreated in a solution of 1,000 ppb riboflavin in 1 M sodium perchlorate. This pretreatment was achieved simply by performing ac voltammetric scans in the presence of riboflavin prior to exposure to TNT or DNT. Note that three distinct redox peaks can now be seen at concentrations of 10 ppb of TNT without background subtraction. While the DNT does not show increased peak currents there appears to be a shift to combine the riboflavin peak with some contributing electrons from the first DNT peak which could explain reported enhanced reduction in environmental samples.

**Figure 3.**
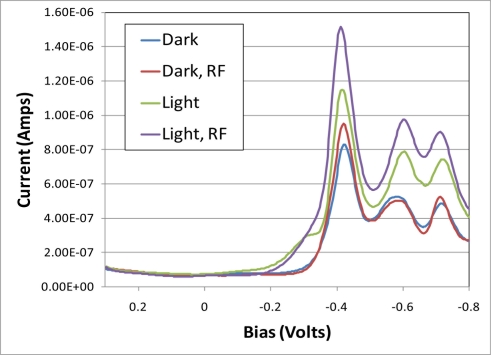
AC voltammograms are presented of electrode response based on exposure to light and electrode pretreatment with riboflavin. These samples all contained both 1,000 ppb TNT and 1,000 ppb riboflavin. Traces marked with RF were pretreated by running three ac voltammograms with 1,000 ppb riboflavin before exposure to TNT. Samples were also measure either in a closed faraday cage to block light or exposed to light. The lowest peak current at ∼−0.45 V is TNT not exposed to light nor pretreated with riboflavin (“Dark”). The highest peak current is exposed to light and pretreated with riboflavin (“Light, RF”).

Figure 3 shows the ac voltammograms of the electrode response to TNT while controlling exposure to riboflavin and light. Each scan is an average of three separate experiments. While each of these scans included equal concentrations of TNT and riboflavin, 1,000 ppb each, there were two main differences. One was whether the electrochemical cell was exposed to light or not and if the electrode was first pretreated with riboflavin or if the riboflavin and TNT were added simultaneously.

**Figure 4.**
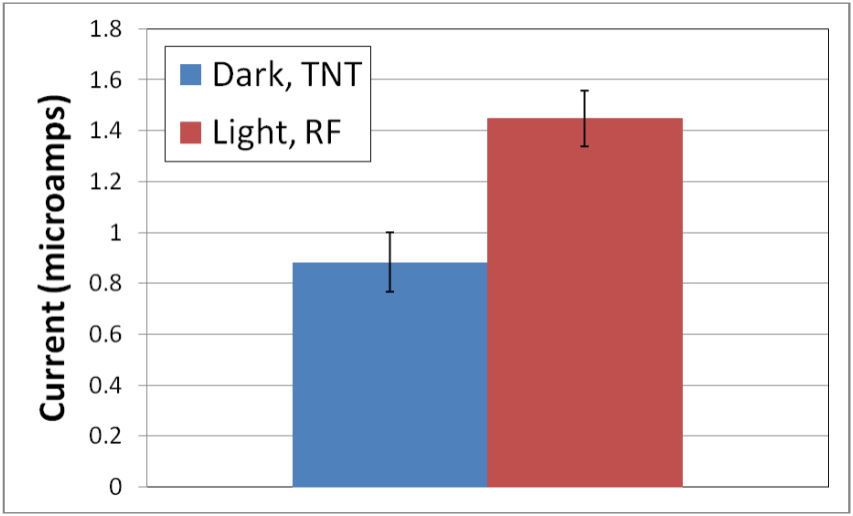
This bar graph compares the reproducibility and enhancement of redox currents of TNT (at the peak current near −0.4 Volts) when exposed to light and riboflavin pretreatment. These are an average and the error bars show a standard deviation of six replicate electrodes. The enhancement is about 60% with the normal sensor having a peak current of 0.88 (σ = 0.12) microamps and the enhanced at 1.45 (σ = 0.11) microamps.

In the data indicated with the labels “RF” in Figure 3, the electrodes were first exposed to 1,000 ppb riboflavin and the ac voltammograms swept three times prior to the addition of 1,000 ppb TNT. The other two data series were exposed to TNT and riboflavin simultaneously. Note that the samples exposed to light and pretreated with riboflavin showed a significant enhancement over measurements performed in a similar matter to previous reports (the trace labeled “Dark”). Figure 4 shows this enhancement and its reproducibility by plotting the average and standard deviation of six independent experiments for both the fully enhanced (“Light, RF”) and the control (“Dark”). The shoulder on the voltammogram labeled “Light” appears to be due to riboflavin.

**Figure 5.**
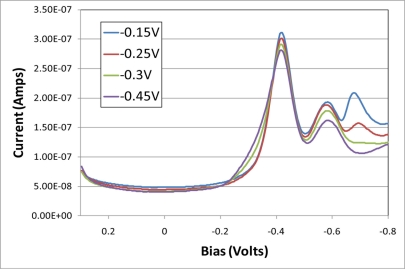
AC voltammograms collected after holding poised potential for 10 min at −0.15, −0.25, −0.30 and −0.45 V. Note that while the first redox peak had little change in peak current the two subsequent peaks were much more significantly affected.

Poised potential experiments were performed to determine if irreversible reduction of the TNT in the electrochemical cell could be achieved with the assistance of riboflavin. The total volume of electrolyte was reduced to 2 mL in these experiments and each potential was held for 10 min followed by collecting an AC voltammogram. Figure 5 shows an example of this experiment. While the first redox peak showed only a slight change in total current the two subsequent peaks were diminished to an increasingly greater extent. Without riboflavin present all three redox peaks decreased by less than 10% under the poised potential experimental conditions.

## 4. Conclusions

This data suggests that riboflavin aids in the reduction of TNT, and thus makes TNT easier to detect and remediate in aqueous environments. While reproducibility can be a challenge, problems can be mitigated using mercaptohexanol, SAM-modified working electrodes. Exposing riboflavin working electrodes to broadband light enhanced redox peaks by 60% over the techniques similar to those previously reported. Further work is necessary to verify that these results would translate to environmental samples. It is possible that there would be compounds in the real world samples that could interfere with the electrochemistry. This data indicates that riboflavin, under these conditions, is capable of transferring electrons to nitroaromatics. Since the redox potential of riboflavin is significantly closer to zero volts, the energy required to reduce the riboflavin and thus reduce the nitroaromatic is significantly less. These results were dramatic enough to explain long term enhancement of bioremediation for nitro aromatics in the environment containing high levels of riboflavin as well as enhance the limit of detection in electrochemically-based TNT sensing.
